# Daratumumab plus bortezomib, cyclophosphamide, and dexamethasone in Asian patients with newly diagnosed AL amyloidosis: subgroup analysis of ANDROMEDA

**DOI:** 10.1007/s00277-023-05090-z

**Published:** 2023-03-02

**Authors:** Kenshi Suzuki, Ashutosh D. Wechalekar, Kihyun Kim, Chihiro Shimazaki, Jin Seok Kim, Takayuki Ikezoe, Chang-Ki Min, Fude Zhou, Zhen Cai, Xiaonong Chen, Shinsuke Iida, Nagaaki Katoh, Tomoaki Fujisaki, Ho-Jin Shin, NamPhuong Tran, Xiang Qin, Sandra Y. Vasey, Brenda Tromp, Brendan M. Weiss, Raymond L. Comenzo, Efstathios Kastritis, Jin Lu

**Affiliations:** 1 Department of Hematology, Japanese Red Cross Medical Center, Tokyo, Japan; 2 Division of Medicine, Faculty of Medical Sciences, University College London and the Royal Free London NHS Foundation Trust, London, UK; 3 Department of Medicine, Samsung Medical Center, Sungkyunkwan University School of Medicine, Seoul, South Korea; 4 Department of Hematology, Japan Community Health Care Organization, Kyoto Kuramaguchi Medical Center, Kyoto, Japan; 5 Yonsei University College of Medicine, Severance Hospital, Seoul, Korea; 6 Department of Hematology, Fukushima Medical School, Fukushima, Japan; 7 Seoul St. Mary’s Hospital, Seoul, Korea; 8 Department of Medicine, Peking University First Hospital, Renal Division, Beijing, China; 9 College of Medicine, The First Affiliated Hospital of Zhejiang University, Hangzhou, Zhejiang China; 10 Department of Nephrology, Ruijin Hospital Affiliated to Shanghai Jiao Tong University, Shanghai, China; 11 Department of Hematology and Oncology, Nagoya City University Institute of Medical and Pharmaceutical Sciences, Nagoya, Japan; 12 Department of Medicine (Neurology and Rheumatology), Shinshu University School of Medicine, Matsumoto, Nagano, Japan; 13 Matsuyama Red Cross Hospital, Matsuyama, Japan; 14 Department of Internal Medicine, Pusan National University Hospital, Busan, South Korea; 15 Janssen Research & Development, LLC, Los Angeles, CA USA; 16 Janssen Research & Development, LLC, Spring House, PA USA; 17 Janssen Biologics B.V., Leiden, The Netherlands; 18 Division of Hematology/Oncology, John C. Davis Myeloma and Amyloid Program, Tufts Medical Center, Boston, MA USA; 19 Department of Clinical Therapeutics, National and Kapodistrian University of Athens, School of Medicine, Athens, Greece; 20 Collaborative Innovation Center of Hematology, Peking University People’s Hospital, Peking University Institute of Hematology, National Clinical Research Center for Hematologic Disease, Beijing, China

**Keywords:** Light-chain amyloidosis, Daratumumab, Asian, Body weight, Efficacy, Safety

## Abstract

Subcutaneous daratumumab plus bortezomib/cyclophosphamide/dexamethasone (VCd; D-VCd) improved outcomes versus VCd for patients with newly diagnosed immunoglobulin light-chain (AL) amyloidosis in the phase 3 ANDROMEDA study. We report a subgroup analysis of Asian patients (Japan; Korea; China) from ANDROMEDA. Among 388 randomized patients, 60 were Asian (D-VCd, *n* = 29; VCd, *n* = 31). At a median follow-up of 11.4 months, the overall hematologic complete response rate was higher for D-VCd versus VCd (58.6% vs. 9.7%; odds ratio, 13.2; 95% confidence interval [CI], 3.3–53.7; *P* < 0.0001). Six-month cardiac and renal response rates were higher with D-VCd versus VCd (cardiac, 46.7% vs. 4.8%; *P* = 0.0036; renal, 57.1% vs. 37.5%; *P* = 0.4684). Major organ deterioration progression-free survival (MOD-PFS) and major organ deterioration event-free survival (MOD-EFS) were improved with D-VCd versus VCd (MOD-PFS: hazard ratio [HR], 0.21; 95% CI, 0.06–0.75; *P* = 0.0079; MOD-EFS: HR, 0.16; 95% CI, 0.05–0.54; *P* = 0.0007). Twelve deaths occurred (D-VCd, *n* = 3; VCd, *n* = 9). Twenty-two patients had baseline serologies indicating prior hepatitis B virus (HBV) exposure; no patient experienced HBV reactivation. Although grade 3/4 cytopenia rates were higher than in the global safety population, the safety profile of D-VCd in Asian patients was generally consistent with the global study population, regardless of body weight. These results support D-VCd use in Asian patients with newly diagnosed AL amyloidosis. ClinicalTrials.gov Identifier: NCT03201965.

**Supplementary Information:**

The online version contains supplementary material available at 10.1007/s00277-023-05090-z.

## Introduction

Immunoglobulin light-chain (AL) amyloidosis is a rare disorder caused by clonal expansion of CD38^+^ plasma cells that produce immunoglobulin light chains that misfold and aggregate into insoluble amyloid fibrils [1]. Deposition of amyloid fibrils in vital organs, most commonly the heart and kidney, can result in severe and life-threatening organ dysfunction [1]. Approved therapies for AL amyloidosis treatment are lacking and standard of care involves therapies targeting plasma cells that were developed for multiple myeloma (MM); the most commonly used regimen for newly diagnosed patients in Europe is bortezomib/cyclophosphamide/dexamethasone (VCd) [2–4]. Although reports on the use of bortezomib-based regimens for AL amyloidosis in Japan, Korea, and China are limited [5–10], a study by Shimazaki et al. demonstrated that bortezomib-based therapies are widely used in Japan [11]. Rapid and deep hematologic responses are critical for optimal AL amyloidosis treatment. Although outcomes have improved with the use of novel MM therapies, particularly bortezomib-based therapies, more effective and tolerable therapies are needed [2–4, 12].

Daratumumab is a human IgGκ monoclonal antibody targeting CD38 with a direct on-tumor [13–16] and immunomodulatory [17–19] mechanism of action. Based on positive efficacy and safety results from clinical trials, intravenous daratumumab (DARA IV) 16 mg/kg and subcutaneous daratumumab (DARA SC) 1800 mg are approved in many countries as monotherapy and in combination with standard-of-care regimens for newly diagnosed MM and relapsed or refractory MM [20–22]. Consistent efficacy and safety with the global study population were seen in Asian patients in the phase 3 POLLUX and ALCYONE studies of DARA IV–containing regimens [23, 24]. In the phase 3 OCTANS and LEPUS studies, which enrolled patients at sites in Asia, efficacy and safety results of DARA IV–containing regimens were also consistent [25, 26]. Additionally, efficacy, pharmacokinetics, and safety of DARA SC in Asian patients in the phase 3 COLUMBA study were consistent with the global study population, regardless of patient body weight [27].

In relapsed or refractory AL amyloidosis, daratumumab has demonstrated an acceptable safety profile and encouraging efficacy in terms of hematologic response rates and improvement in organ function [28–34]. In the phase 3 ANDROMEDA study, safety and efficacy of DARA SC plus VCd (D-VCd) are being evaluated in patients with newly diagnosed AL amyloidosis. Results from the safety run-in of ANDROMEDA demonstrated that D-VCd was well tolerated [35]. In the primary analysis of the randomized portion of the study, D-VCd resulted in a significantly higher hematologic complete response (CR) rate versus VCd (53.3% vs. 18.1%; *P* < 0.0001) [36]. Deeper and more rapid hematologic responses with D-VCd versus VCd were associated with delayed major organ deterioration, hematologic progression, or death (major organ deterioration progression-free survival [MOD-PFS]) and improved organ responses at 6 months.

To determine whether the efficacy and safety results of D-VCd in Asian patients with newly diagnosed AL amyloidosis are similar to those observed in the global study population, we performed a post hoc analysis of Asian patients (enrolled at sites in Japan, Korea, and China) from ANDROMEDA.

## Patients and methods

### Patients

A total of 60 Asian patients (enrolled at sites in Japan [*n* = 28], Korea [*n* = 20], or China [*n* = 12]) from ANDROMEDA (enrollment occurred between May 2018 and August 2019) were included in this analysis. Complete eligibility criteria have been published previously [36]. Briefly, eligible patients were ≥ 18 years of age with a histopathologic diagnosis of systemic AL amyloidosis (≥ 1 involved organ) and measurable hematologic disease with no prior therapy. See Online Resource 1 (Supplementary Methods) for additional details.

### Study design and treatment

ANDROMEDA is a randomized, open-label, active-controlled, phase 3 study. Patients were randomized (1:1) to receive VCd with or without DARA SC (daratumumab 1800 mg co-formulated with recombinant human hyaluronidase PH20 [2000 U/mL; ENHANZE^® ^drug delivery technology, Halozyme, Inc., San Diego, CA, USA]). All patients received bortezomib 1.3 mg/m^2^ subcutaneously, cyclophosphamide 300 mg/m^2^ orally or intravenously [500 mg maximum weekly dose], and dexamethasone 40 mg orally or intravenously once weekly for 6 cycles of 28 days each. DARA SC was administered by manual injection over approximately 5 min weekly in cycles 1 and 2, every 2 weeks in cycles 3–6, and every 4 weeks thereafter until disease progression, until the start of subsequent therapy, or for a maximum of 24 cycles from the start of the study, whichever occurred first. The median follow-up period was 11.4 months. See Online Resource 1 (Supplementary Methods) for additional details.

### Endpoints and assessments

The primary endpoint was overall hematologic CR rate at the time of clinical cutoff, as assessed by the independent review committee that was blinded to treatment assignment. Key secondary endpoints included MOD-PFS, major organ deterioration event-free survival (MOD-EFS), organ response rate [37, 38], organ response rate at 6 months, overall survival, hematologic CR at 6 months, hematologic very good partial response or better (≥ VGPR) rate, time to and duration of hematologic CR, and safety. See the Online Resource 1 (Supplementary Methods) for additional details.

### Evaluation and statistical analyses

The intent-to-treat (ITT) population included all randomized patients. The safety population included all patients who received ≥ 1 dose of trial treatment. Between-group difference for overall hematologic CR rate in the ITT population was tested using a stratified Cochran–Mantel–Haenszel test, and corresponding common odds ratios, 95% confidence intervals (CIs), and *P* values were reported. For Asian patients, the *P* value was derived from a chi-square test. See the Online Resource 1 (Supplementary Methods) for additional details.

### Study oversight

The study was approved by independent ethics committees or institutional review boards at each site and was conducted in accordance with the Declaration of Helsinki and the International Conference on Harmonisation Good Clinical Practice guidelines. All patients provided written informed consent. The study design and analyses were devised by the investigators and sponsor, and study data were collected by the investigators and their research teams. Final data analysis and verification of accuracy were conducted by Janssen. Investigators were not restricted by confidentiality agreements and had full access to all data. Writing assistance was funded by Janssen Global Services, LLC. The study was sponsored by Janssen Research & Development, LLC, and was registered at ClinicalTrials.gov (NCT03201965).

## Results

### Patients and treatments

A total of 388 patients were randomized in ANDROMEDA (D-VCd, *n* = 195; VCd, *n* = 193) [36]; 60 (15.5%) patients were included in the Asian cohort analysis (D-VCd, *n* = 29; VCd, *n* = 31), including 28 patients from 9 sites in Japan (D-VCd, *n* = 15; VCd, *n* = 13), 20 patients from 5 sites in Korea (D-VCd, *n* = 8; VCd, *n* = 12), and 12 patients from 4 sites in China (D-VCd, *n* = 6; VCd, *n* = 6). Baseline patient demographics and clinical characteristics of the Asian cohort were generally well balanced between arms and consistent with the ITT population (Table 1) [36]. In the Asian cohort, median age was 66 (range, 42–82) years, median body weight was 61.7 (range, 38.0–92.0) kg, and median time since diagnosis was 44 (range, 11–304) days. Only 2 patients (both in the VCd arm) in the Asian cohort had a body weight of > 85 kg. The median baseline difference between involved and uninvolved free light chain was 170 (range, 4–9983) mg/L. Thirty-six patients (60.0%) had ≥ 2 organs involved; 70.0% of patients had heart involvement, and 58.3% had kidney involvement. Most patients (71.7%) were classified as cardiac stage II or higher. In the Asian cohort, a higher percentage of D-VCd patients was classified as cardiac stage I and a lower percentage was classified as cardiac stage II compared with VCd patients. Compared to the ITT population [36], median body weight in the Asian cohort was lower, and the percentage of patients with cardiac stage I was higher.

**Table 1:** Demographic and baseline disease characteristics (ITT population)^a^

	ANDROMEDA ITT population [36]		Asian cohort	
	D-VCd (*n* = 195)	VCd (*n* = 193)	D-VCd (*n* = 29)	VCd (*n* = 31)
Age				
Median (range), years	62 (34–87)	64 (35–86)	62 (42–82)	68 (46–79)
< 65 years, *n* (%)	108 (55.4)	97 (50.3)	17 (58.6)	10 (32.3)
≥ 65 years, *n* (%)	87 (44.6)	96 (49.7)	12 (41.4)	21 (67.7)
Male, *n* (%)	108 (55.4)	117 (60.6)	17 (58.6)	21 (67.7)
Body weight, kg				
Median (range)	73.0 (41.5–141.5)	70.0 (38.0–134.6)	61.3 (41.5–81.0)	62.2 (38.0–92.0)
≤ 65 kg, *n* (%)	62 (31.8)	74 (38.3)	23 (79.3)	22 (71.0)
> 65 to 85 kg, *n* (%)	96 (49.2)	74 (38.3)	6 (20.7)	7 (22.6)
> 85 kg, *n* (%)	37 (19.0)	45 (23.3)	0	2 (6.5)
ECOG performance status, *n* (%)^b^				
0	90 (46.2)	71 (36.8)	18 (62.1)	15 (48.4)
1	86 (44.1)	106 (54.9)	10 (34.5)	14 (45.2)
2	19 (9.7)	16 (8.3)	1 (3.4)	2 (6.5)
AL isotype, *n* (%)^c^				
Lambda	158 (81.0)	149 (77.2)	22 (75.9)	26 (83.9)
Kappa	37 (19.0)	44 (22.8)	7 (24.1)	5 (16.1)
Median (range) baseline dFLC, mg/L (range)	200 (2–4749)	186 (1–9983)	140 (4–901)	267 (23–9983)
Median (range) bone marrow plasma cells, %	10 (1–50)	10 (0–55)	5.9 (1–28)	6.4 (1–24)
Median (range) time since amyloidosis diagnosis, days	48 (8–1611)	43 (5–1102)	49 (11–236)	43 (11–304)
Involved organs				
Median (range)	2 (1–5)	2 (1–6)	2 (1–3)	2 (1–5)
Distribution, *n* (%)				
Heart	140 (71.8)	137 (71.0)	19 (65.5)	23 (74.2)
Kidney	115 (59.0)	114 (59.1)	18 (62.1)	17 (54.8)
Liver	15 (7.7)	16 (8.3)	2 (6.9)	3 (9.7)
Other^d^	127 (65.1)	124 (64.2)	12 (41.4)	17 (54.8)
Cardiac stage, *n* (%)^e^				
I	47 (24.1)	43 (22.3)	12 (41.4)	5 (16.1)
II	76 (39.0)	80 (41.5)	5 (17.2)	12 (38.7)
IIIA	70 (35.9)	64 (33.2)	12 (41.4)	12 (38.7)
IIIB^f^	2 (1.0)	6 (3.1)	0	2 (6.5)
Renal stage, *n* (%)^g,h^				
I	107/193 (55.4)	101/193 (52.3)	16/29 (55.2)	16/31 (51.6)
II	67/193 (34.7)	74/193 (38.3)	11/29 (37.9)	10/31 (32.3)
III	19/193 (9.8)	18/193 (9.3)	2/29 (6.9)	5/31 (16.1)
Creatinine clearance, *n* (%)				
< 60 mL/min	69 (35.4)	62 (32.1)	14 (48.3)	10 (32.3)
≥ 60 mL/min	126 (64.6)	131 (67.9)	15 (51.7)	21 (67.7)
From countries that typically offer transplant for patients with AL amyloidosis, *n* (%)				
Yes	147 (75.4)	146 (75.6)	23 (79.3)	25 (80.6)
No	48 (24.6)	47 (24.4)	6 (20.7)	6 (19.4)
Median (range) N-terminal pro–B-type natriuretic peptide, ng/L	1388.6 (51–10,182)	1746 (51–12,950)	681.1 (51–7241)	2022.1 (83–11,688)
Median (range) estimated glomerular filtration rate, mL/min/1.73 m^2^	77.8 (21–126)	76.2 (20–121)	84.8 (26–113)	74.7 (23–109)
Cytogenetic risk profile^h^				
* n*	155	166	29	30
Standard risk, *n* (%)	138 (89.0)	147 (88.6)	26 (89.7)	26 (86.7)
High risk, *n* (%)	17 (11.0)	19 (11.4)	3 (10.3)	4 (13.3)

*ITT*, intent-to-treat; *D-VCd*, daratumumab subcutaneous plus bortezomib/cyclophosphamide/dexamethasone; *VCd*, bortezomib/cyclophosphamide/dexamethasone; *ECOG*, Eastern Cooperative Oncology Group; *AL*, immunoglobulin light-chain; *dFLC*, difference between involved and uninvolved free light chain

^a^The ITT population included all randomized patients

^b^ECOG performance status is scored on a scale from 0 to 5, with 0 indicating no symptoms and higher scores indicating increasing disability

^c^Based on immunofixation or light-chain measurement

^d^Includes gastrointestinal tract, lung, peripheral nervous system, autonomic nervous system, and soft tissue

^e^Based on the European modification of the Mayo Clinic Cardiac Staging System [3], cardiac stage was based on 2 biomarker risk factors: N-terminal pro–B-type natriuretic peptide and high-sensitivity cardiac troponin T assessed via central laboratory

^f^All patients were stage IIIA at screening; however, some converted to IIIB at cycle 1, day 1 (results by central laboratory were only made available after cycle 1, day 1)

^g^Renal stage is derived based on the combination of estimated glomerular filtration rate and proteinuria[37, 38]

^h^Patients with high-risk cytogenetic abnormalities had a t(4;14), t(14;16), and/or del17p abnormality assessed locally via fluorescence in situ hybridization or t(4;14) and/or del17p abnormality assessed locally via karyotyping. In China, bone marrow aspirates and/or biopsies were sent to a central laboratory if the local laboratory could not conduct the fluorescence in situ hybridization assessment. Patients with standard-risk cytogenetic abnormalities had an absence of high-risk cytogenetic abnormalities

In the ITT population, 193 and 188 patients in the D-VCd and VCd arms, respectively, received ≥ 1 dose of trial treatment [36]; all patients in the Asian cohort received ≥ 1 treatment dose. At the time of clinical data cutoff for the primary analysis (February 14, 2020), 52 (26.7%) D-VCd patients and 68 (35.2%) VCd patients in the ITT population had discontinued treatment (Table 2) [36]. In the Asian cohort, 4 (13.8%) D-VCd patients and 9 (29.0%) VCd patients had discontinued treatment (Table 2). In the global safety population, median duration of treatment was 9.6 (range, 0.03–21.2) months with D-VCd and 5.3 (range, 0.03–7.3) months with VCd [36], and the median number of cycles received was 11 (range, 1–23) with D-VCd and 6 (range, 1–6) with VCd. In the Asian cohort, median duration of treatment was 9.2 (range, 1.0–21.2) months with D-VCd and 5.3 (range, 0.03–6.1) months with VCd, and median number of cycles received was 11 (range, 2–23) with D-VCd and 6 (range, 1–6) with VCd. In the global safety population, 159 (82.4%) and 121 (64.4%) patients received 6 treatment cycles in the D-VCd and VCd arms, respectively, and in the D-VCd arm, 149 (77.2%) patients continued single-agent DARA SC after completing the first 6 cycles [36]. In the Asian cohort, 25 (86.2%) and 22 (71.0%) patients completed 6 treatment cycles in the D-VCd and VCd arms, respectively, and in the D-VCd arm, all 25 (86.2%) patients continued single-agent DARA SC after completing the first 6 cycles. Consistent with the global safety population [36], the incidence of dose reductions was similar between treatment arms (global safety population: cyclophosphamide, 17.6% vs. 13.8%; bortezomib, 25.9% vs. 19.7%; dexamethasone, 27.5% vs. 27.7%; Asian cohort: cyclophosphamide, 24.1% vs. 32.3%; bortezomib, 24.1% vs. 29.0%; dexamethasone, 17.2% vs. 12.9%). Dose reductions were not permitted for DARA SC.

**Table 2:** Patient disposition (ITT population)^a^

	ANDROMEDA ITT population [36]		Asian cohort	
	D-VCd (*n* = 195)	VCd (*n* = 193)	D-VCd (*n* = 29)	VCd (*n* = 31)
Discontinued study treatment, *n* (%)	52 (26.7)	68 (35.2)	4 (13.8)	9 (29.0)
Death	20 (10.3)	14 (7.3)	2 (6.9)	4 (12.9)
Received ASCT	12 (6.2)	3 (1.6)	0	0
Adverse event	8 (4.1)	8 (4.1)	1 (3.4)	1 (3.2)
Subsequent therapy	5 (2.6)	23 (11.9)	0	0
Patient withdrawal	3 (1.5)	7 (3.6)	1 (3.4)	2 (6.5)
Physician decision	1 (0.5)	1 (0.5)	0	0
Progressive disease	2 (1.0)	11 (5.7)	0	1 (3.2)
Other	1 (0.5)	1 (0.5)	0	1 (3.2)

*ITT*, intent-to-treat; *D-VCd*, daratumumab subcutaneous plus bortezomib/cyclophosphamide/dexamethasone; *VCd*, bortezomib/cyclophosphamide/dexamethasone; *ASCT*, autologous stem cell transplant

^a^The ITT population included all randomized patients

### Efficacy

At a median follow-up of 11.4 (range, 0.03–21.3) months for the ITT population, the overall hematologic CR rate was higher with D-VCd versus VCd in the ITT population (53.3% vs. 18.1%; odds ratio, 5.1; 95% CI, 3.2–8.2; *P* < 0.0001) [36] and Asian cohort (58.6% vs. 9.7%; odds ratio, 13.2; 95% CI, 3.3–53.7; *P* < 0.0001; Table 3). Hematologic CR rates at 6 months were consistent with overall hematologic CR rates in the ITT population (D-VCd, 49.7% vs. VCd, 14.0%; odds ratio, 6.1; 95% CI, 3.7–10.0; *P* < 0.0001) [36] and Asian cohort (D-VCd, 58.6% vs. VCd, 9.7%; odds ratio, 13.2; 95% CI, 3.3–53.7; *P* < 0.0001). Among patients who achieved hematologic CR, median time to hematologic CR was 1.97 months with D-VCd versus 2.79 months with VCd in the global study population [36] and 1.94 months with D-VCd versus 2.83 months with VCd in the Asian cohort. The rate of hematologic ≥ VGPR and overall hematologic response rate were higher with D-VCd versus VCd in the ITT population (≥ VGPR, 78.5% vs. 49.2%; overall response rate, 91.8% vs. 76.7%) [36] and Asian cohort (≥ VGPR, 93.1% vs. 61.3%; overall response rate, 100.0% vs. 93.5%; Table 3). Among patients who achieved hematologic ≥ VGPR, median time to hematologic ≥ VGPR was 0.56 months with D-VCd versus 0.82 months with VCd in the global study population and 0.53 months with D-VCd versus 0.99 months with VCd in the Asian cohort.

**Table 3:** Summary of overall confirmed hematologic responses^a^ and cardiac and renal reponses^b^ at 6 months (ITT population)^c^

	ANDROMEDA ITT population [36]		Asian cohort	
	D-VCd (*n* = 195)	VCd (*n* = 193)	D-VCd (*n* = 29)	VCd (*n* = 31)
ORR, *n* (%)	179 (91.8)	148 (76.7)	29 (100.0)	29 (93.5)
CR^d^	104 (53.3)	35 (18.1)	17 (58.6)	3 (9.7)
≥ VGPR	153 (78.5)	95 (49.2)	27 (93.1)	19 (61.3)
VGPR	49 (25.1)	60 (31.1)	10 (34.5)	16 (51.6)
PR	26 (13.3)	53 (27.5)	2 (6.9)	10 (32.3)
No response, *n* (%)	8 (4.1)	38 (19.7)	0	1 (3.2)
PD, *n* (%)	0	0	0	0
NE, *n* (%)	8 (4.1)	7 (3.6)	0	1 (3.2)
Cardiac response at 6 months, *n*/*N*^e^ (%)				
Overall	49/118 (41.5)	26/117 (22.2)	7/15 (46.7)	1/21 (4.8)
Cardiac stage I	NE	NE	NE	NE
Cardiac stage II	28/55 (50.9)	16/54 (29.6)	3/3 (100.0)	0/9
Cardiac stage IIIA/B	21/63 (33.3)	10/63 (15.9)	4/12 (33.3)	1/12 (8.3)
Renal response at 6 months, *n*/*N*^f^ (%)				
Overall	62/117 (53.0)	27/113 (23.9)	12/21 (57.1)	6/16 (37.5)
Cardiac stage I	24/36 (66.7)	9/34 (26.5)	6/9 (66.7)	1/4 (25.0)
Cardiac stage II	25/44 (56.8)	17/46 (37.0)	3/3 (100.0)	2/7 (28.6)
Cardiac stage IIIA/B	14/37 (37.8)	5/33 (15.2)	3/9 (33.3)	3/5 (60.0)

*ITT*, intent-to-treat; *D-VCd*, daratumumab subcutaneous plus bortezomib/cyclophosphamide/dexamethasone; *VCd*, bortezomib/cyclophosphamide/dexamethasone; *ORR*, overall response rate; *CR*, complete response; *VGPR*, very good partial response; *PR*, partial response; *PD*, progressive disease; *NE*, not evaluable; *FLC*, free light chain; *iFLC*, involved free light chain; *dFLC*, difference between involved and uninvolved free light chain; *NT-proBNP*, N-terminal pro–B-type natriuretic peptide; *NYHA* New York Heart Association

^a^Hematologic response was assessed centrally in the ITT population, which included all randomized patients

^b^Organ response–evaluable population (patients with measurable organ involvement); organ responses were assessed by a blinded independent review committee. Responses were assessed via previously validated criteria[37, 38]

^c^The ITT population included all randomized patients

^d^CR was based on consensus criteria with clarifications, which required confirmation by the independent review committee that was blinded to treatment assignment. CR was defined as negative immunofixation and FLC ratio normalization without confirmation [37], reduction in absolute iFLC (to ≤ 20 mg/L) [42], and dFLC (to < 10 mg/L)[4]

^e^Cardiac response evaluable was defined as patients with baseline NT-proBNP value ≥ 650 ng/L or baseline NYHA class 3 or 4. In addition, patients must have received ≥ 1 administration of study treatment and have ≥ 1 post-baseline NT-proBNP measurement (if baseline NT-proBNP ≥ 650 ng/L) or NYHA function evaluation (if baseline NYHA class 3 or 4)

^f^Renal response evaluable was defined as patients with baseline urine protein > 0.5 g/day. In addition, patients must have received ≥ 1 administration of study treatment and have ≥ 1 post-baseline urine protein (g/day) measurement

Among those evaluable for cardiac response, the 6-month cardiac response rate was higher with D-VCd versus VCd in the global study population (41.5% vs. 22.2%; *P* = 0.0029) [36] and Asian cohort (46.7% vs. 4.8%; *P* = 0.0036; Table 3). Among those evaluable for renal response, the 6-month renal response rate was also higher with D-VCd versus VCd in the global study population (53.0% vs. 23.9%; *P* < 0.0001) [36] and Asian cohort (57.1% vs. 37.5%; *P* = 0.4684). Cardiac and renal response rates at 6 months were generally higher with D-VCd regardless of baseline cardiac stage in both the global study population and Asian cohort (Table 3).

**Fig. 1:**
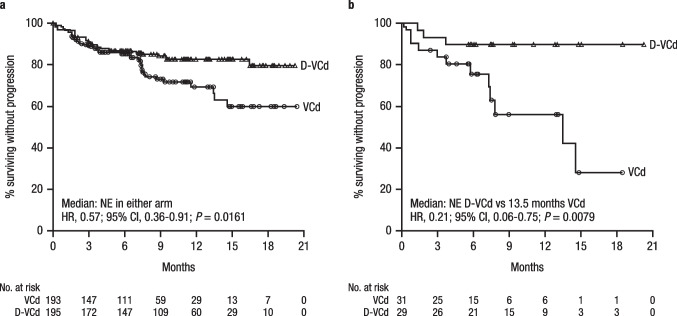
MOD-PFS^a,b^ of **a** the global ITT population and **b** the Asian cohort. MOD-PFS, major organ deterioration progression-free survival; ITT, intent-to-treat; D-VCd, daratumumab subcutaneous plus bortezomib/cyclophosphamide/dexamethasone; VCd, bortezomib/cyclophosphamide/dexamethasone; NE, not estimable; HR, hazard ratio; CI, confidence interval; IPCW, inverse probability of censoring weighting. ^a^Because of the small number of Asian patients, an IPCW analysis method was not applicable to analyze MOD-PFS, and MOD-PFS was based on independent review committee assessment after adjusting for dependent censoring due to subsequent non-cross-resistant anti-plasma cell therapy. MOD-PFS was defined as the time from randomization to any of the following events (whichever occurred first): death, clinical manifestation of cardiac or renal failure, or hematologic progression. ^b^Evaluated in the ITT population, which included all randomized patients

**Fig. 2:**
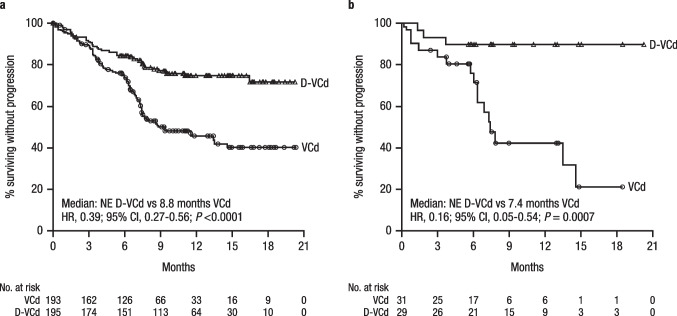
MOD-EFS^a,b^ of **a** the global ITT population and **b** the Asian cohort. MOD-EFS, major organ deterioration event-free survival; ITT, intent-to-treat; D-VCd, daratumumab subcutaneous plus bortezomib/cyclophosphamide/dexamethasone; VCd, bortezomib/cyclophosphamide/dexamethasone; NE, not estimable; HR, hazard ratio; CI, confidence interval; IPCW, inverse probability of censoring weighting. ^a^Because of the small number of Asian patients, an IPCW analysis method was not applicable to analyze MOD-EFS, and MOD-EFS was based on independent review committee assessment after adjusting for dependent censoring due to subsequent non-cross-resistant anti-plasma cell therapy. MOD-EFS was defined as hematologic progression, end-stage cardiac or renal disease, initiation of subsequent non-cross-resistant anti-plasma cell therapy, or death, whichever came first. ^b^Evaluated in the ITT population, which included all randomized patients

MOD-PFS was improved with D-VCd versus VCd in the ITT population (median: not estimable [NE] in either arm; hazard ratio [HR], 0.57; 95% CI, 0.36–0.91; *P* = 0.0161; Fig. 1a) and in the Asian cohort (median: NE vs. 13.5 months; HR, 0.21; 95% CI, 0.06–0.75; *P* = 0.0079; Fig. 1b). In the Asian cohort, 3 events of hematologic progression, major organ deterioration, or death occurred with D-VCd versus 12 events with VCd. MOD-EFS was also improved with D-VCd versus VCd in the ITT population (median, NE vs. 8.8 months; HR, 0.39; 95% CI, 0.27–0.56; *P* < 0.0001; Fig. 2a) [36] and in the Asian cohort (median, NE vs. 7.4 months; HR, 0.16; 95% CI, 0.05–0.54; *P* = 0.0007; Fig. 2b). In the Asian cohort, 3 events of hematologic progression, major organ deterioration, initiation of subsequent therapy, or death occurred with D-VCd versus 16 events with VCd. Median time to next treatment was NE with D-VCd versus 10.4 months with VCd in the ITT population (HR, 0.20; 95% CI, 0.12–0.32; *P* < 0.0001) and NE in either treatment arm in the Asian cohort (HR, 0.10; 95% CI, 0.01–0.79; *P* = 0.0069). Overall survival results remained immature at the time of this analysis.

Efficacy results of the Asian cohort based on baseline body weight are reported in the Supplementary Materials (Online Resource 1 [Supplementary Results] and Online Resource 2 [Supplementary Table 1]).

### Safety

Any grade treatment-emergent adverse events (TEAEs) occurred in almost all patients in the global safety population [36] and Asian cohort (Table 4). Grade 3/4 TEAEs occurred in 113 (58.5%) patients with D-VCd and 108 (57.4%) patients with VCd in the global safety population and 19 (65.5%) patients with D-VCd and 25 (80.6%) patients with VCd in the Asian cohort (Table 4). When adjusted for exposure to study treatment, the incidence rate of any grade and grade 3/4 TEAEs was lower with D-VCd versus VCd in the global safety population [36]. In the Asian cohort, the exposure-adjusted incidence rate of grade 3/4 TEAEs was also lower with D-VCd versus VCd, whereas the exposure-adjusted incidence rate of any grade TEAEs was higher with D-VCd versus VCd (Table 4).

**Table 4:** Summary of safety results (safety population)^a^

	ANDROMEDA safety population [36]		Asian cohort	
	D-VCd (*n* = 193)	VCd (*n* = 188)	D-VCd (*n* = 29)	VCd (*n* = 31)
Any grade TEAE, *n* (%)	189 (97.9)	185 (98.4)	29 (100.0)	30 (96.8)
Any grade infection	127 (65.8)	101 (53.7)	19 (65.5)	20 (64.5)
Any grade cardiac disorder	63 (32.6)	41 (21.8)	6 (20.7)	6 (19.4)
Grade 3/4 TEAE, *n* (%)	113 (58.5)	108 (57.4)	19 (65.5)	25 (80.6)
Grade 3/4 infection	32 (16.6)	19 (10.1)	6 (20.7)	4 (12.9)
Grade 3/4 cardiac disorder	22 (11.4)	18 (9.6)	2 (6.9)	4 (12.9)
Any grade TEAE, EAIR^b^	154.23	217.92	353.08	224.91
Grade 3/4 TEAE, EAIR^b^	10.55	18.96	11.17	33.86
TEAEs leading to treatment discontinuation	8 (4.1)	8 (4.3)	1 (3.4)	1 (3.2)
TEAEs resulting in death	22 (11.4)	15 (8.0)	3 (10.3)	4 (12.9)
SAE, *n* (%)	83 (43.0)	68 (36.2)	10 (34.5)	14 (45.2)
Serious infection	31 (16.1)	16 (8.5)	2 (6.9)	3 (9.7)

*D-VCd*, daratumumab subcutaneous plus bortezomib/cyclophosphamide/dexamethasone; *VCd*, bortezomib/cyclophosphamide/dexamethasone; *TEAE*, treatment-emergent adverse event; *EAIR*, exposure-adjusted incidence rate; *SAE*, serious adverse event

^a^The safety population included patients who received ≥ 1 administration of study treatment

^b^Events per 100 patient-months at risk

The most common any grade (> 25% of patients in any group) and grade 3/4 (≥ 5% of patients in any group) TEAEs are summarized in Table 5. Rates of grade 3/4 lymphopenia (D-VCd, 34.5% and VCd, 32.3%), neutropenia (10.3% and 3.2%), and leukopenia (6.9% and 3.2%) were higher in the Asian cohort compared to the global safety population [36]. In the global safety population, any grade and grade 3/4 infections occurred at a higher rate with D-VCd versus VCd [36]. In the Asian cohort, any grade infections were reported at a similar rate between D-VCd and VCd, while a higher rate of grade 3/4 infections was reported with D-VCd versus VCd (Table 4). In the global safety population, 52 (D-VCd, 25 [13.0%]; VCd, 27 [14.4%]) patients had baseline serologies consistent with prior exposure to hepatitis B virus (HBV). In the Asian cohort, 22 (D-VCd, 10 [34.5%]; VCd, 12 [38.7%]) patients had baseline serologies consistent with prior HBV exposure. No patient in the study had documented HBV reactivation. The rate of grade 3/4 cardiac disorders was similar between D-VCd versus VCd in the global safety population; the rate was lower with D-VCd versus VCd in the Asian cohort (D-VCd, 6.9% and VCd, 12.9%; Table 4). Of the patients who continued to receive single-agent DARA SC, 12 (6.2%) patients in the global safety population [36] and 1 (3.4%) patient in the Asian cohort experienced cardiac disorders from cycle 7 and beyond.

**Table 5:** Most common any grade (> 25%) and grade 3/4 (≥ 5%) TEAEs (safety population)^a^

	ANDROMEDA safety population [36]				Asian cohort			
	D-VCd (*n* = 193)		VCd (*n* = 188)		D-VCd (*n* = 29)		VCd (*n* = 31)	
TEAE, *n* (%)	Any grade	Grade 3/4	Any grade	Grade 3/4	Any grade	Grade 3/4	Any grade	Grade 3/4
Hematologic	86 (44.6)	35 (18.1)	77 (41.0)	33 (17.6)	15 (51.7)	12 (41.4)	19 (61.3)	14 (45.2)
Anemia	47 (24.4)	8 (4.1)	44 (23.4)	9 (4.8)	6 (20.7)	1 (3.4)	6 (19.4)	3 (9.7)
Lymphopenia	36 (18.7)	25 (13.0)	28 (14.9)	19 (10.1)	12 (41.4)	10 (34.5)	11 (35.5)	10 (32.3)
Thrombocytopenia	33 (17.1)	6 (3.1)	22 (11.7)	5 (2.7)	4 (13.8)	1 (3.4)	8 (25.8)	3 (9.7)
Neutropenia	21 (10.9)	10 (5.2)	12 (6.4)	5 (2.7)	5 (17.2)	3 (10.3)	4 (12.9)	1 (3.2)
Leukopenia	11 (5.7)	2 (1.0)	7 (3.7)	2 (1.1)	6 (20.7)	2 (6.9)	3 (9.7)	1 (3.2)
Infections	127 (65.8)	32 (16.6)	101 (53.7)	19 (10.1)	19 (65.5)	6 (20.7)	20 (64.5)	4 (12.9)
Upper respiratory tract infection	50 (25.9)	1 (0.5)	21 (11.2)	1 (0.5)	7 (24.1)	1 (3.4)	6 (19.4)	0
Pneumonia	21 (10.9)	15 (7.8)	12 (6.4)	8 (4.3)	2 (6.9)	2 (6.9)	4 (12.9)	3 (9.7)
Herpes zoster	10 (5.2)	0	12 (6.4)	2 (1.1)	4 (13.8)	0	7 (22.6)	2 (6.5)
Diarrhea	69 (35.8)	11 (5.7)	57 (30.3)	7 (3.7)	12 (41.4)	3 (10.3)	13 (41.9)	2 (6.5)
Peripheral edema	69 (35.8)	6 (3.1)	68 (36.2)	11 (5.9)	3 (10.3)	0	4 (12.9)	2 (6.5)
Constipation	66 (34.2)	3 (1.6)	54 (28.7)	0	12 (41.4)	1 (3.4)	10 (32.3)	0
Peripheral sensory neuropathy	60 (31.1)	5 (2.6)	37 (19.7)	4 (2.1)	5 (17.2)	0	2 (6.5)	1 (3.2)
Fatigue	52 (26.9)	8 (4.1)	53 (28.2)	6 (3.2)	2 (6.9)	1 (3.4)	4 (12.9)	1 (3.2)
Nausea	52 (26.9)	3 (1.6)	52 (27.7)	0	6 (20.7)	1 (3.4)	7 (22.6)	0
Asthenia	31 (16.1)	4 (2.1)	20 (10.6)	2 (1.1)	2 (6.9)	0	3 (9.7)	2 (6.5)
Hypokalemia	24 (12.4)	3 (1.6)	28 (14.9)	10 (5.3)	4 (13.8)	2 (6.9)	5 (16.1)	3 (9.7)
Cardiac failure^b^	18 (9.3)	12 (6.2)	14 (7.4)	9 (4.8)	3 (10.3)	2 (6.9)	4 (12.9)	3 (9.7)
Increased alanine aminotransferase	18 (9.3)	5 (2.6)	10 (5.3)	1 (0.5)	5 (17.2)	2 (6.9)	1 (3.2)	0
Syncope	14 (7.3)	10 (5.2)	12 (6.4)	12 (6.4)	1 (3.4)	1 (3.4)	3 (9.7)	3 (9.7)
Hypoalbuminemia	9 (4.7)	1 (0.5)	11 (5.9)	5 (2.7)	5 (17.2)	1 (3.4)	5 (16.1)	3 (9.7)
Hypercholesterolemia	6 (3.1)	1 (0.5)	5 (2.7)	2 (1.1)	5 (17.2)	1 (3.4)	4 (12.9)	2 (6.5)

*TEAE*, treatment-emergent adverse event; *D-VCd*, daratumumab subcutaneous plus bortezomib/cyclophosphamide/dexamethasone; *VCd*, bortezomib/cyclophosphamide/dexamethasone

^a^The safety population included patients who received ≥ 1 administration of study treatment

^b^Includes overall and congestive cardiac failure

Serious adverse events (SAEs) occurred in 83 (43.0%) patients with D-VCd and 68 (36.2%) patients with VCd in the global safety population [36] and 10 (34.5%) patients with D-VCd and 14 (45.2%) patients with VCd in the Asian cohort (Table 4). The most common SAE was pneumonia in the global safety population (global: D-VCd, 7.3%; VCd, 4.8% [36]; Asian cohort: D-VCd, 0; VCd, 9.7%); cardiac failure (including overall and congestive cardiac failure) was the most common SAE in the Asian cohort (global: D-VCd, 6.2%; VCd, 4.3%; Asian cohort: D-VCd, 10.3%; VCd, 12.9%).

TEAEs leading to treatment discontinuation occurred in 8 patients in each arm in the global safety population (D-VCd, 4.1% and VCd, 4.3%) [36] and 1 patient in each arm of the Asian cohort (3.4% and 3.2%; Table 4). Infections leading to treatment discontinuation of any study treatment occurred in 2 (1.0%) patients with D-VCd and 1 (0.5%) patient with VCd in the global safety population; no infections led to treatment discontinuation in the Asian cohort. TEAEs resulting in death in the global safety population occurred in 22 (11.4%) patients with D-VCd and 15 (8.0%) patients with VCd (Table 4). TEAEs resulting in death in the Asian cohort occurred in 3 (10.3%) patients with D-VCd (cardiac failure [*n* = 2], sudden death [*n* = 1]) and 4 (12.9%) patients with VCd (cardiac failure [*n* = 1], myocardial infarction [*n* = 1], sinus node dysfunction [*n* = 1], and ischemic stroke [*n* = 1]).

In the global safety population, deaths occurred in 27 (14.0%) patients with D-VCd and 29 (15.4%) patients with VCd [36]; deaths during the first 6 months occurred in 25 (13.0%) and 20 (10.6%) patients, respectively. In the Asian cohort, deaths occurred in 3 (10.3%) patients with D-VCd and 9 (29.0%) patients with VCd; deaths during the first 6 months occurred in 3 (10.3%) and 5 (16.1%) patients, respectively. Adverse events were the most common primary cause of death in the global safety population and Asian cohort (global: D-VCd, 11.9%; VCd, 7.4% [36]; Asian cohort: D-VCd, 6.9%; VCd, 9.7%). Disease progression as the primary cause of death was less frequent with D-VCd versus VCd (global: D-VCd, 1.0%; VCd, 4.8% [36]; Asian cohort: D-VCd, 3.4%; VCd, 9.7%), as were other reasons (global: D-VCd, 1.0%; VCd, 2.7% [36]; Asian cohort: D-VCd, 0%; VCd, 9.7%).

Fourteen (7.3%) patients in the global safety population and 3 (10.3%) patients in the Asian cohort experienced systemic administration-related reactions to DARA SC, all of which were grade 1 or 2 [36]. In the global safety population, 54 (28.0%) patients in the D-VCd arm and 45 (23.9%) patients in the VCd arm experienced local injection-site reactions; 21 (10.9%) patients in the D-VCd arm experienced local injection-site reactions related to DARA SC, all of which were grade 1 or 2 [36]. No Asian patient experienced local injection-site reactions.

Safety results of the Asian cohort based on baseline body weight are reported in the Supplementary Materials (Online Resource 1 [Supplementary Results] and Online Resources 3–5 [Supplementary Tables 2–4]).

## Discussion

In this post hoc subgroup analysis of Asian patients enrolled in ANDROMEDA, a higher hematologic CR rate and deeper and more rapid hematologic responses were observed with D-VCd versus VCd; results were generally consistent across body weight subgroups. Improved MOD-PFS, MOD-EFS, and cardiac and renal response rates at 6 months were also observed in the Asian cohort, with improved MOD-PFS and 6-month organ response rates seen across body weight subgroups. In the Asian cohort, cardiac and renal response rates at 6 months were generally higher with D-VCd versus VCd, regardless of baseline cardiac stage. These results indicate that the addition of daratumumab to VCd elicits deeper responses and prolongs MOD-PFS and MOD-EFS compared with VCd alone in Asian patients. The efficacy results presented here for the Asian cohort overall and by baseline body weight are consistent with those from the global ANDROMEDA population [36].

Of note, although the hematologic ORR and ≥ VGPR rates in the VCd arm of the ANDROMEDA Asian cohort (ORR, 93.5%; ≥ VGPR, 61.3%) were in line with those from other published reports for bortezomib-containing regimens in Asian patients (ORR, 66.2–90.0%; ≥ VGPR, 54.2–75.0%), a lower proportion of patients in the ANDROMEDA Asian cohort achieved hematologic CR with VCd (CR, 9.7%) compared to these other reports (CR, 36.1–60.0%) [6, 39, 40]. However, such cross-study comparisons should be interpreted with caution due to differences in study designs, treatment regimens, and patient populations.

D-VCd demonstrated an acceptable safety profile in Asian patients that was generally consistent with the global safety population from ANDROMEDA and the known safety profile of the individual components [20, 21, 36, 41]. Consistent with the Asian subgroup analysis of COLUMBA, higher rates of grade 3/4 cytopenias were observed in the Asian cohort of ANDROMEDA versus the global safety population [27, 36]; in the current study, rates were similar between treatment arms. Higher rates of grade 3/4 cytopenias in the Asian cohort may be attributed to lower median baseline body weight in this cohort versus the global safety population. Notably, despite higher rates of grade 3/4 cytopenias in the Asian cohort, rates of grade 3/4 and serious infections in the Asian cohort were similar to or lower than those in the global safety population [36]. Rates of grade 3/4 infections were higher with D-VCd versus VCd in both the global safety population [36] and Asian cohort, which may be attributed to the longer treatment duration and longer adverse event collection period in the D-VCd arm. When adjusted for exposure to study treatment, incidence rates of grade 3/4 TEAEs were lower with D-VCd versus VCd in the global safety population [36] and in the Asian cohort overall and across body weight subgroups. The rate of serious pneumonia, a common SAE associated with daratumumab [20–22], was similar between the Asian cohort and global safety population [36], and no patient in the Asian cohort experienced serious pneumonia with D-VCd. Although 36.7% of Asian patients had baseline serologies consistent with prior HBV exposure, in this study, no Asian patient had documented HBV reactivation; these findings were consistent with observations in the global safety population. Patients in the Asian cohort did not experience an increased rate of grade 3/4 cardiac disorders compared to the global safety population, and rates of grade 3/4 cardiac disorders in the Asian cohort were lower with D-VCd, including in the lower body weight subgroup. The rate of TEAEs resulting in death was higher with D-VCd versus VCd in the global safety population but was similar between treatment arms in the Asian cohort. Consistent with the global safety population [36], administration-related reactions were infrequent and mild in the Asian cohort. No local injection-site reactions related to DARA SC were observed in Asian patients.

There are several limitations of this post hoc analysis. The imbalance in cardiac stage between treatment groups in the Asian cohort may have impacted the magnitude of the efficacy differences observed between D-VCd and VCd in favor of the D-VCd group. Additionally, this analysis was limited by data immaturity and by the relatively low patient numbers in the Asian cohort.

Results of this subgroup analysis complement those reported for the Asian subgroup analysis of COLUMBA in relapsed or refractory MM [27]. In Asian patients in COLUMBA, DARA SC 1800 mg flat dose was comparable to DARA IV 16 mg/kg, and no new safety concerns were observed. Efficacy and safety results with DARA SC were consistent with those observed in the global COLUMBA population, regardless of patient body weight [27].

The addition of DARA SC to VCd was superior to VCd alone in Asian patients, resulting in deeper and more rapid hematologic responses and improved organ responses. Treatment with D-VCd improved clinical outcomes, including MOD-PFS and MOD-EFS, versus VCd alone in Asian patients. Although this post hoc subgroup analysis was limited by data immaturity and a small sample size, efficacy and safety of D-VCd in Asian patients overall and of low body weight were generally consistent with those of the global ANDROMEDA population [36]. These results support the use of D-VCd in Asian patients with newly diagnosed AL amyloidosis.

## Supplementary Information

Below is the link to the electronic supplementary material.

Supplementary file1 (PDF 242 KB)

## Data Availability

The data sharing policy of Janssen Pharmaceutical Companies of Johnson & Johnson is available at https://www.janssen.com/clinical-trials/transparency. As noted on this site, requests for access to the study data can be submitted through the Yale Open Data Access (YODA) Project site at http://yoda.yale.edu.
