# Supplementary material for: Daratumumab plus bortezomib, cyclophosphamide, and dexamethasone in Asian patients with newly diagnosed AL amyloidosis: subgroup analysis of ANDROMEDA
Source: Ann Hematol. 2023 Mar 2;102(4):863–76. doi: 10.1007/s00277-023-05090-z (PMC9998577; doi:10.1007/s00277-023-05090-z)
Supplement: Supplementary file 1 — Supplementary file1 (PDF 242 KB) [file 277_2023_5090_MOESM1_ESM.pdf]

Supplementary Information for Suzuki et al. Daratumumab plus bortezomib, cyclophosphamide, and dexamethasone in Asian patients with newly diagnosed AL amyloidosis: subgroup analysis of ANDROMEDA

Online Resource 1

Supplementary Methods

Patients

Patients were required to have an Eastern Cooperative Oncology Group performance status of ≤2, a cardiac stage (European modification of the Mayo Clinic Cardiac Staging System 2004) [1] of I–IIIA, and an estimated glomerular filtration rate of ≥20 mL/min/1.73 m^2. Patients with a previous or current diagnosis of symptomatic multiple myeloma or evidence of a severe cardiovascular condition (including N-terminal pro–B-type natriuretic peptide >8,500 ng/L, systolic blood pressure <90 mm Hg, or a New York Heart Association classification of stage IIIB or IV heart failure at screening) were excluded.

Study Design and Treatment

For patients who were >70 years of age; who were underweight (body mass index <18.5 kg/m^2); or who had hypervolemia, poorly controlled diabetes mellitus, or prior intolerance to steroid therapy, dexamethasone could be administered at a dose of 20 mg weekly at the discretion of their physician.

Randomization was stratified according to cardiac stage (I, II, or IIIA based on the European modification of the Mayo Clinic Cardiac Staging System) [1], renal function (creatinine clearance ≥60 or <60 mL/min), and availability of transplant in local country (countries that do or do not typically offer transplant for patients with AL amyloidosis).

Endpoints and Assessments

Hematologic complete response (CR) was defined as an involved free light chain (FLC) level of less than the upper limit of normal with negative serum and urine immunofixation; there was no requirement for a normalized uninvolved FLC or FLC ratio to determine CR [2-4].

Major organ deterioration progression-free survival (MOD-PFS) was defined as the time from randomization to death, clinical manifestation of end-stage cardiac or renal failure, or hematologic progression, whichever came first. Major

organ deterioration event-free survival (MOD-EFS) was defined as hematologic progression, clinical manifestation of end-stage cardiac or renal disease, initiation of subsequent non–cross-resistant anti–plasma cell therapy, or death, whichever came first.

Disease status was evaluated once every 4 weeks in cycles 1 through 6 and once every 8 weeks in cycle 7 and beyond until hematologic progression, major organ deterioration, death, or withdrawal from the study.

All patients were tested locally for hepatitis B surface antigen (HBsAg), antibodies to hepatitis B surface antigen (anti-HBs), and antibodies to hepatitis B core antigen (anti-HBc) at screening, with the exception of patients who were tested within 3 months prior to the first dose of study treatment. Patients had baseline serologies consistent with prior hepatitis B virus (HBV) exposure if they were positive for anti-HBs, HBsAg, or anti-HBc at screening. Patients who were seropositive for hepatitis B (positive for HBsAg) were excluded from the study. In patients with serologic evidence of resolved HBV infection (ie, patients who were negative for HBsAg but positive for anti-HBc and/or anti-HBs) at screening, real-time polymerase chain reaction (PCR) was performed to measure HBV DNA levels, and patients who were PCR positive for HBV DNA were excluded from the study. PCR testing was not required for patients with serologic findings suggestive of HBV vaccination (anti-HBs positivity as the only serologic marker) and a known history of prior HBV vaccination. Patients who had a history of HBV infection were closely monitored during and following receipt of study treatment for clinical and laboratory signs of HBV reactivation.

Evaluation and Statistical Analyses

Continuous variables were summarized using descriptive statistics including mean, standard deviation, median, and range. Categorical variables were summarized using frequency and percentage. For time-to-event variables, the Kaplan–Meier method was used for descriptive summaries.

Exploratory subgroup analyses were conducted in the Asian cohort to assess the efficacy and safety of daratumumab subcutaneous plus bortezomib/cyclophosphamide/dexamethasone (D-VCd) versus bortezomib/cyclophosphamide/dexamethasone (VCd) based on baseline body weight.

Supplementary Results

Efficacy body weight subgroup analyses

When patients in the Asian cohort were assessed by baseline body weight, hematologic CR rates overall and at 6 months for D-VCd versus VCd were 54.5% versus 18.2%, respectively, in the ≤55-kg subgroup, 41.7% versus 0% in the >55-65–kg subgroup, and 100.0% versus 11.1% in the >65-kg subgroup (**Online Resource 2** [**Supplementary Table 1**]). Among Asian patients who achieved hematologic CR, median time to hematologic CR for D-VCd versus VCd was 2.07 versus 2.37 months, respectively, in the ≤55-kg subgroup and 1.94 versus 3.71 months in the >65-kg subgroup. Median time to hematologic CR for D-VCd in the >55-65–kg subgroup was 1.94 months; no Asian patients achieved hematologic CR with VCd in this subgroup. The rate of hematologic ≥VGPR was higher with D-VCd versus VCd in all body weight subgroups, and overall hematologic response rate was 100.0% in both arms in all body weight subgroups, except with VCd in the 55-65–kg subgroup (81.8%; **Online Resource 2** [**Supplementary Table 1**]). Among Asian patients who achieved hematologic ≥VGPR, median time to hematologic ≥VGPR for D-VCd versus VCd was 0.56 versus 0.53 months, respectively, in the ≤55-kg subgroup, 0.39 versus 1.91 months in the >55-65–kg subgroup, and 0.54 versus 1.46 months in the >65-kg subgroup. Among patients evaluable for cardiac response, the 6-month cardiac response rate with D-VCd versus VCd was 50.0% versus 11.1% in the ≤55-kg subgroup, 33.3% versus 0% in the >55-65–kg subgroup, and 66.7% versus 0% in the >65-kg subgroup (**Online Resource 2** [**Supplementary Table 1**]). Among patients evaluable for renal response, the 6-month renal response rate was 71.4% versus 33.3% in the ≤55-kg subgroup, 44.4% versus 60.0% in the >55-65–kg subgroup, and 60.0% versus 20.0% in the >65-kg subgroup (**Online Resource 2** [**Supplementary Table 1**]).

In the Asian cohort, 3 events of hematologic progression, major organ deterioration, or death occurred with D-VCd (2 patients in the ≤55-kg subgroup and 1 patient in the >55-65–kg subgroup) versus 12 events with VCd (6 patients in the ≤55-kg subgroup, 4 patients in the 55-65–kg subgroup, and 2 patients in the >65-kg subgroup). Median MOD-PFS was NE with D-VCd versus 7.4 months with VCd in the ≤55-kg subgroup (HR, 0.21; 95% CI, 0.04-1.09; P=0.0423), NE with D-VCd versus 14.6 months with VCd in the >55-65–kg subgroup (HR, 0.29; 95% CI, 0.03-2.79; P=0.253), and NE in either arm in the >65-kg subgroup (there were no events in the D-VCd arm in this subgroup). In the Asian cohort, 3 events of hematologic progression, major organ deterioration, initiation of subsequent therapy, or death occurred with D-VCd (2 patients in the ≤55-kg subgroup and 1 patient in the >55-65–kg subgroup) versus 16 events with VCd (6 patients in the ≤55-kg subgroup, 8 patients in the >55-65–kg subgroup, and 2 patients in the >65-kg

subgroup).

Safety body weight subgroup analyses

In the Asian cohort, patients with baseline body weight >55-65 kg had higher rates of grade 3/4 TEAEs compared to those with baseline body weight >65 kg. When adjusted for exposure to study treatment, the incidence rate of any grade and grade 3/4 TEAEs was lower with D-VCd versus VCd in the global safety population [5]; similar results were seen in the Asian cohort overall and in body weight subgroups, with the exception of a higher exposure–adjusted incidence rate of any grade TEAEs with D-VCd versus VCd in the Asian cohort overall and in the >65-kg subgroup (**Table 4**, **Online Resource 3** [**Supplementary Table 2**], and **Online Resource 4** [**Supplementary Table 3**]). Similar to the overall Asian cohort, rates of grade 3/4 lymphopenia, neutropenia, and leukopenia were higher in patients in the Asian cohort with lower body weight (≤55-kg and >55-65–kg subgroups) compared to the overall global safety population (**Table 5** and **Online Resource 5** [**Supplementary Table 4**]) [5]. In the Asian cohort, any grade infections were reported at a similar rate between D-VCd and VCd, while a higher rate of grade 3/4 infections was reported with D-VCd versus VCd; a similar pattern was observed across body weight subgroups (**Table 4** and **Online Resource 3** [**Supplementary Table 2**]). Of the patients who continued to receive single-agent DARA SC, 1 (3.4%) patient in the Asian cohort (from the >55-65–kg subgroup) experienced cardiac disorders from cycle 7 and beyond. As in the Asian cohort overall, lower SAE rates were observed with D-VCd versus VCd across body weight subgroups (**Table 4** and **Online Resource 3** [**Supplementary Table 2**]). TEAEs leading to treatment discontinuation occurred in 1 patient in each arm of the Asian cohort (3.4% and 3.2%); the 2 Asian patients were in the ≤55-kg subgroup (**Table 4** and **Online Resource 3** [**Supplementary Table 2**]). TEAEs resulting in death in the Asian cohort occurred in 3 (10.3%) patients with D-VCd (cardiac failure in 1 patient in the ≤55-kg subgroup and 1 patient in the >55-65–kg subgroup; sudden death in 1 patient in the ≤55-kg subgroup) and 4 (12.9%) patients with VCd (cardiac failure, myocardial infarction, and sinus node dysfunction in 1 patient each in the ≤55-kg subgroup; ischemic stroke in 1 patient in the >55-65–kg subgroup). In the Asian cohort, deaths occurred in 3 (10.3%) patients with D-VCd (2 patients in the ≤55-kg subgroup and 1 patient in the >55-65–kg subgroup) and 9 (29.0%) patients with VCd (5 patients in the ≤55-kg subgroup, 3 patients in the >55-65–kg subgroup, and 1 patient in the >65-kg subgroup). Three (10.3%) patients in the Asian cohort (1 patient in the ≤55-kg subgroup and 2 patients in the >55-65–kg subgroup) experienced systemic administration–related reactions to DARA SC, all of which were grade 1 or 2.

Online Resource 2

Supplementary Table 1. Summary of Overall Confirmed Hematologic Responses[a] and Cardiac and Renal Reponses[b] at 6 Months by Baseline Body Weight (ITT Population)[c]

	**ANDROMEDA ITT population [5]**		**Asian cohort**		**Asian ≤55 kg**		**Asian >55-65 kg**		**Asian >65 kg**	
	**D-VCd (n=195)**	**VCd (n=193)**	**D-VCd (n=29)**	**VCd (n=31)**	**D-VCd (n=11)**	**VCd (n=11)**	**D-VCd (n=12)**	**VCd (n=11)**	**D-VCd (n=6)**	**VCd (n=9)**
ORR, n (%)	179 (91.8)	148 (76.7)	29 (100.0)	29 (93.5)	11 (100.0)	11 (100.0)	12 (100.0)	9 (81.8)	6 (100.0)	9 (100.0)
CR[d]	104 (53.3)	35 (18.1)	17 (58.6)	3 (9.7)	6 (54.5)	2 (18.2)	5 (41.7)	0	6 (100.0)	1 (11.1)
≥VGPR	153 (78.5)	95 (49.2)	27 (93.1)	19 (61.3)	10 (90.9)	6 (54.5)	11 (91.7)	5 (45.5)	6 (100.0)	8 (88.9)
VGPR	49 (25.1)	60 (31.1)	10 (34.5)	16 (51.6)	4 (36.4)	4 (36.4)	6 (50.0)	5 (45.5)	0	7 (77.8)
PR	26 (13.3)	53 (27.5)	2 (6.9)	10 (32.3)	1 (9.1)	5 (45.5)	1 (8.3)	4 (36.4)	0	1 (11.1)
No response, n (%)	8 (4.1)	38 (19.7)	0	1 (3.2)	0	0	0	1 (9.1)	0	0
PD, n (%)	0	0	0	0	0	0	0	0	0	0
NE, n (%)	8 (4.1)	7 (3.6)	0	1 (3.2)	0	0	0	1 (9.1)	0	0
Cardiac response at 6 months, n/N[e] (%)										
Overall	49/118 (41.5)	26/117 (22.2)	7/15 (46.7)	1/21 (4.8)	3/6 (50.0)	1/9 (11.1)	2/6 (33.3)	0/6	2/3 (66.7)	0/6
Cardiac stage I	NE	NE	NE	NE	–	–	–	–	–	–
Cardiac stage II	28/55 (50.9)	16/54 (29.6)	3/3 (100.0)	0/9	–	–	–	–	–	–
Cardiac stage IIIA/B	21/63 (33.3)	10/63 (15.9)	4/12 (33.3)	1/12 (8.3)	–	–	–	–	–	–
Renal response at 6 months, n/N[f] (%)										
Overall	62/117 (53.0)	27/113 (23.9)	12/21 (57.1)	6/16 (37.5)	5/7 (71.4)	2/6 (33.3)	4/9 (44.4)	3/5 (60.0)	3/5 (60.0)	1/5 (20.0)
Cardiac stage I	24/36 (66.7)	9/34 (26.5)	6/9 (66.7)	1/4 (25.0)	–	–	–	–	–	–
Cardiac stage II	25/44 (56.8)	17/46 (37.0)	3/3 (100.0)	2/7 (28.6)	–	–	–	–	–	–
Cardiac stage IIIA/B	14/37 (37.8)	5/33 (15.2)	3/9 (33.3)	3/5 (60.0)	–	–	–	–	–	–

ITT, intent-to-treat; D-VCd, daratumumab subcutaneous plus bortezomib/cyclophosphamide/dexamethasone; VCd, bortezomib/cyclophosphamide/dexamethasone; ORR, overall response rate; CR, complete response; VGPR, very good partial response; PR, partial response; PD, progressive disease; NE, not evaluable; FLC, free light chain; iFLC, involved free light chain; dFLC, difference between involved and uninvolved free light chain; NT-proBNP, N-terminal pro–B-type natriuretic peptide; NYHA New York Heart Association.

[a]Hematologic response was assessed centrally in the ITT population, which included all randomized patients.

[b]Organ response–evaluable population (patients with measurable organ involvement); organ responses were assessed by a blinded independent review committee. Responses were assessed via previously validated criteria [6,7].

[c]The ITT population included all randomized patients.

[d]CR was based on consensus criteria with clarifications, which required confirmation by the independent review committee that was blinded to treatment assignment. CR was defined as negative immunofixation and FLC ratio normalization without confirmation [6], reduction in absolute iFLC (to ≤20 mg/L) [3], and dFLC (to <10 mg/L) [4].
[e]Cardiac response evaluable was defined as patients with baseline NT-proBNP value ≥650 ng/L or baseline NYHA class 3 or 4. In addition, patients must have received ≥1 administration of study treatment and have ≥1 post-baseline NT-proBNP measurement (if baseline NT-proBNP ≥650 ng/L) or NYHA function evaluation (if baseline NYHA class 3 or 4).
[f]Renal response evaluable was defined as patients with baseline urine protein >0.5 g/day. In addition, patients must have received ≥1 administration of study treatment and have ≥1 post-baseline urine protein (g/day) measurement.

Online Resource 3

Supplementary Table 2. Summary of Safety Results by Baseline Body Weight (Safety Population)[a]

	**ANDROMEDA safety population [5]**		**Asian cohort**		**Asian ≤55 kg**		**Asian >55-65 kg**		**Asian >65 kg**	
	**D-VCd (n=193)**	**VCd (n=188)**	**D-VCd (n=29)**	**VCd (n=31)**	**D-VCd (n=11)**	**VCd (n=11)**	**D-VCd (n=12)**	**VCd (n=11)**	**D-VCd (n=6)**	**VCd (n=9)**
Any grade TEAE, n (%)	189 (97.9)	185 (98.4)	29 (100.0)	30 (96.8)	11 (100.0)	11 (100.0)	12 (100.0)	11 (100.0)	6 (100.0)	8 (88.9)
Any grade infection	127 (65.8)	101 (53.7)	19 (65.5)	20 (64.5)	7 (63.6)	7 (63.6)	8 (66.7)	7 (63.6)	4 (66.7)	6 (66.7)
Any grade cardiac disorder	63 (32.6)	41 (21.8)	6 (20.7)	6 (19.4)	1 (9.1)	5 (45.5)	5 (41.7)	1 (9.1)	0	0
Grade 3/4 TEAE, n (%)	113 (58.5)	108 (57.4)	19 (65.5)	25 (80.6)	5 (45.5)	10 (90.9)	10 (83.3)	9 (81.8)	4 (66.7)	6 (66.7)
Grade 3/4 infection	32 (16.6)	19 (10.1)	6 (20.7)	4 (12.9)	2 (18.2)	1 (9.1)	2 (16.7)	1 (9.1)	2 (33.3)	2 (22.2)
Grade 3/4 cardiac disorder	22 (11.4)	18 (9.6)	2 (6.9)	4 (12.9)	0	3 (27.3)	2 (16.7)	1 (9.1)	0	0
Any grade TEAE, EAIR[b]	154.23	217.92	353.08	224.91	334.81	523.14	380.47	492.37	338.19	88.87
Grade 3/4 TEAE, EAIR[b]	10.55	18.96	11.17	33.86	5.11	42.27	34.24	42.27	9.29	20.78
TEAEs leading to treatment discontinuation	8 (4.1)	8 (4.3)	1 (3.4)	1 (3.2)	1 (9.1)	1 (9.1)	0	0	0	0
TEAEs resulting in death	22 (11.4)	15 (8.0)	3 (10.3)	4 (12.9)	2 (18.2)	3 (27.3)	1 (8.3)	1 (9.1)	0	0
SAE, n (%)	83 (43.0)	68 (36.2)	10 (34.5)	14 (45.2)	4 (36.4)	7 (63.6)	5 (41.7)	5 (45.5)	1 (16.7)	2 (22.2)
Serious infection	31 (16.1)	16 (8.5)	2 (6.9)	3 (9.7)	0	0	1 (8.3)	1 (9.1)	1 (16.7)	2 (22.2)

D-VCd, daratumumab subcutaneous plus bortezomib/cyclophosphamide/dexamethasone; VCd, bortezomib/cyclophosphamide/dexamethasone; TEAE, treatment-emergent adverse event; EAIR, exposure-adjusted incidence rate; SAE, serious adverse event.

[a]The safety population included patients who received ≥1 administration of study treatment.

[b]Events per 100 patient-months at risk.

Online Resource 4

Supplementary Table 3. Exposure-adjusted Incidence Rates of the Most Common Any Grade (>25%) and Grade 3/4 (>10%) TEAEs (Safety Population)[a]

	**ANDROMEDA safety population [5]**				**Asian cohort**				**Asian ≤55 kg**				**Asian >55–65 kg**				**Asian >65 kg**			
	**D-VCd (n=193)**		**VCd (n=188)**		**D-VCd (n=29)**		**VCd (n=31)**		**D-VCd (n=11)**		**VCd (n=11)**		**D-VCd (n=12)**		**VCd (n=11)**		**D-VCd (n=6)**		**VCd (n=9)**	
**TEAE**	**Any grade**	**Grade 3/4**	**Any grade**	**Grade 3/4**	**Any grade**	**Grade 3/4**	**Any grade**	**Grade 3/4**	**Any grade**	**Grade 3/4**	**Any grade**	**Grade 3/4**	**Any grade**	**Grade 3/4**	**Any grade**	**Grade 3/4**	**Any grade**	**Grade 3/4**	**Any grade**	**Grade 3/4**
	Events per 100 patient-months at risk																			
Hematologic	7.35	2.16	12.84	4.34	8.19	5.80	22.87	12.67	4.17	2.88	32.83	11.96	33.74	21.63	23.91	16.03	3.30	1.52	15.39	10.10
Anemia	3.16	0.44	6.42	1.13	2.41	0.37	5.50	2.42	0.89	0	10.59	2.78	5.69	1.21	5.25	2.21	1.49	0	2.34	2.34
Lymphopenia	2.24	1.46	3.74	2.42	5.88	4.57	9.98	8.22	1.83	1.82	8.81	7.79	23.28	16.03	13.74	13.20	3.30	1.52	7.53	4.41
Thrombocytopenia	1.99	0.32	2.82	0.61	1.52	0.36	6.57	2.29	1.83	0	5.08	0	2.38	1.12	4.56	4.46	0	0	10.37	2.22
Neutropenia	1.22	0.55	1.54	0.62	1.99	1.13	3.28	0.75	3.04	1.89	0	0	2.42	1.12	4.88	2.22	0	0	4.99	0
Leukopenia	0.61	0.11	0.88	0.24	2.42	0.74	2.42	0.75	0.90	0.90	2.79	0	7.49	1.12	2.33	2.22	0	0	2.21	0
Infections	14.31	1.84	17.92	2.40	12.51	2.33	23.81	3.10	12.69	1.73	24.27	2.39	14.36	2.40	30.31	2.24	9.77	3.41	18.71	4.69
Upper respiratory tract infection	3.40	0.05	2.74	0.12	2.99	0.36	5.08	0	2.00	0.87	5.79	0	3.72	0	5.10	0	3.74	0	4.51	0
Pneumonia	1.18	0.83	1.49	0.99	0.72	0.72	3.12	2.34	0.83	0.83	2.44	0	1.13	1.13	2.24	2.24	0	0	4.69	4.69
Nasopharyngitis	1.10	0	1.39	0	2.18	0	1.50	0	5.27	0	2.57	0	1.19	0	0	0	0	0	2.15	0
Herpes zoster	0.57	0	1.50	0.24	1.61	0	5.85	1.48	1.76	0	8.16	2.39	1.22	0	4.76	2.16	1.90	0	4.88	0
Cellulitis	0.11	0.05	0.61	0.25	0.36	0.36	0	0	0	0	0	0	0	0	0	0	1.52	1.52	0	0
Cytomegalovirus enterocolitis	0.05	0.05	0	0	0.37	0.37	0	0	0	0	0	0	0	0	0	0	1.56	1.56	0	0
Escherichia bacteremia	0.05	0.05	0	0	0.37	0.37	0	0	0	0	0	0	0	0	0	0	1.55	1.55	0	0
Peripheral edema	5.75	0.33	10.81	1.38	1.17	0	3.19	1.48	2.02	0	5.81	2.55	1.13	0	4.51	2.04	0	0	0	0
Diarrhea	5.26	0.61	8.87	0.88	5.90	1.16	13.34	1.52	5.92	0.84	34.14	2.54	10.95	2.90	4.82	2.18	1.56	0	11.29	0
Constipation	5.07	0.16	8.45	0	7.46	0.37	9.37	0	3.80	0	22.19	0	13.77	1.22	4.67	0	7.80	0	5.42	0
Peripheral sensory neuropathy	4.40	0.27	5.14	0.49	2.15	0	1.54	0.74	1.94	0	2.78	0	2.60	0	0	0	1.92	0	2.20	2.18
Nausea	3.67	0.16	8.10	0	2.52	0.38	6.11	0	1.80	0	20.65	0	4.27	0	4.59	0	1.77	1.77	0	0
Fatigue	3.58	0.44	8.10	0.73	0.77	0.36	3.07	0.73	1.01	0	2.43	0	1.12	1.12	4.42	2.07	0	0	2.29	0
Insomnia	3.13	0	6.95	0.24	2.52	0	3.08	0	3.11	0	2.50	0	2.60	0	4.54	0	1.56	0	2.18	0
Pyrexia	1.53	0	2.03	0.12	3.25	0	5.92	0	2.02	0	12.56	0	4.49	0	2.27	0	4.01	0	4.72	0
Asthenia	1.92	0.22	2.62	0.24	0.77	0	2.34	1.50	2.02	0	9.05	5.23	0	0	0	0	0	0	0	0
Vomiting	1.58	0	2.76	0.12	2.58	0	0.76	0	4.60	0	0	0	2.63	0	2.32	0	0	0	0	0
Hypokalemia	1.39	0.16	3.71	1.26	1.57	0.73	4.06	2.34	0.83	0.83	5.29	2.54	1.21	0	4.93	2.26	3.77	1.55	2.24	2.24
Decreased appetite	1.11	0	3.05	0	2.08	0	6.15	0	0	0	14.26	0	6.26	0	2.21	0	1.77	0	4.93	0
Cardiac failure	0.88	0.54	1.25	0.61	1.11	0.74	3.11	2.25	0.83	0	8.42	4.95	2.52	2.52	2.17	2.17	0	0	0	0
Increased blood creatinine	0.96	0.22	2.08	0.25	0.74	0	4.29	0.76	0	0	2.66	0	1.16	0	2.33	2.33	1.55	0	8.37	0
Rash	0.96	0	1.70	0	1.15	0	0.77	0	2.92	0	0	0	0	0	2.33	0	0	0	0	0
Hyponatremia	0.89	0.27	0.87	0.62	1.13	0.37	0.74	0.74	0.90	0.90	0	0	1.17	0	0	0	1.45	0	2.18	2.17
Syncope	0.79	0.56	1.50	1.50	0.37	0.37	2.26	2.26	0	0	2.41	2.41	1.28	1.28	4.50	4.50	0	0	0	0
Hyperglycemia	0.66	0.22	0.88	0.12	0.72	0.36	2.45	0	0	0	2.82	0	1.12	0	2.32	0	1.46	1.46	2.28	0
Hypoalbuminemia	0.49	0.05	1.39	0.62	1.97	0.36	4.19	2.37	0.85	0.83	8.75	5.46	5.97	0	0	0	0	0	5.43	2.40
Hypertriglyceridemia	0.49	0	0.50	0.12	1.14	0	3.26	0.76	0	0	2.45	0	4.03	0	2.29	0	0	0	5.23	2.40
Increased blood alkaline phosphatase	0.49	0.05	1.38	0.12	1.12	0.36	3.98	0	0.84	0.84	2.54	0	2.55	0	2.09	0	0	0	7.83	0
Acute kidney injury	0.38	0.22	0.87	0.37	0	0	1.51	0.75	0	0	2.45	0	0	0	0	0	0	0	2.29	2.29
Hypercholesterolemia	0.33	0.05	0.62	0.25	2.04	0.37	3.29	1.58	0.91	0	2.44	0	4.30	1.23	2.33	2.29	1.54	0	5.33	2.40
Nephrotic syndrome	0.05	0.05	0	0	0.37	0.37	0	0	0	0	0	0	0	0	0	0	1.57	1.57	0	0
Acute respiratory distress syndrome	0	0	0.12	0.12	0	0	0.75	0.75	0	0	0	0	0	0	0	0	0	0	2.29	2.29

TEAE, treatment-emergent adverse event; D-VCd, daratumumab subcutaneous plus bortezomib/cyclophosphamide/dexamethasone; VCd, bortezomib/cyclophosphamide/dexamethasone.

[a]The safety population included patients who received ≥1 administration of study treatment.

Online Resource 5

Supplementary Table 4. Most Common Any Grade (>25%) and Grade 3/4 (>10%) TEAEs in the Asian Cohort by Baseline Body Weight (Safety Population)[a]

	Asian ≤55 kg				Asian >55–65 kg				Asian >65 kg			
	D-VCd (n=11)		VCd (n=11)		D-VCd (n=12)		VCd (n=11)		D-VCd (n=6)		VCd (n=9)	
TEAE, n (%)	Any grade	Grade 3/4	Any grade	Grade 3/4	Any grade	Grade 3/4	Any grade	Grade 3/4	Any grade	Grade 3/4	Any grade	Grade 3/4
Hematologic	4 (36.4)	3 (27.3)	7 (63.6)	4 (36.4)	9 (75.0)	8 (66.7)	7 (63.6)	6 (54.5)	2 (33.3)	1 (16.7)	5 (55.6)	4 (44.4)
Neutropenia	3 (27.3)	2 (18.2)	0	0	2 (16.7)	1 (8.3)	2 (18.2)	1 (9.1)	0	0	2 (22.2)	0
Lymphopenia	2 (18.2)	2 (18.2)	3 (27.3)	3 (27.3)	8 (66.7)	7 (58.3)	5 (45.5)	5 (45.5)	2 (33.3)	1 (16.7)	3 (33.3)	2 (22.2)
Thrombocytopenia	2 (18.2)	0	2 (18.2)	0	2 (16.7)	1 (8.3)	2 (18.2)	2 (18.2)	0	0	4 (44.4)	1 (11.1)
Leukopenia	1 (9.1)	1 (9.1)	1 (9.1)	0	5 (41.7)	1 (8.3)	1 (9.1)	1 (9.1)	0	0	1 (11.1)	0
Anemia	1 (9.1)	0	3 (27.3)	1 (9.1)	4 (33.3)	1 (8.3)	2 (18.2)	1 (9.1)	1 (16.7)	0	1 (11.1)	1 (11.1)
Infections	7 (63.6)	2 (18.2)	7 (63.6)	1 (9.1)	8 (66.7)	2 (16.7)	7 (63.6)	1 (9.1)	4 (66.7)	2 (33.3)	6 (66.7)	2 (22.2)
Nasopharyngitis	4 (36.4)	0	1 (9.1)	0	1 (8.3)	0	0	0	0	0	1 (11.1)	0
Herpes zoster	2 (18.2)	0	3 (27.3)	1 (9.1)	1 (8.3)	0	2 (18.2)	1 (9.1)	1 (16.7)	0	2 (22.2)	0
Upper respiratory tract infection	2 (18.2)	1 (9.1)	2 (18.2)	0	3 (25.0)	0	2 (18.2)	0	2 (33.3)	0	2 (22.2)	0
Pneumonia	1 (9.1)	1 (9.1)	1 (9.1)	0	1 (8.3)	1 (8.3)	1 (9.1)	1 (9.1)	0	0	2 (22.2)	2 (22.2)
Cellulitis	0	0	0	0	0	0	0	0	1 (16.7)	1 (16.7)	0	0
Cytomegalovirus enterocolitis	0	0	0	0	0	0	0	0	1 (16.7)	1 (16.7)	0	0
Escherichia bacteremia	0	0	0	0	0	0	0	0	1 (16.7)	1 (16.7)	0	0
Diarrhea	5 (45.5)	1 (9.1)	7 (63.6)	1 (9.1)	6 (50.0)	2 (16.7)	2 (18.2)	1 (9.1)	1 (16.7)	0	4 (44.4)	0
Vomiting	4 (36.4)	0	0	0	2 (16.7)	0	1 (9.1)	0	0	0	0	0
Constipation	3 (27.3)	0	6 (54.5)	0	6 (50.0)	1 (8.3)	2 (18.2)	0	3 (50.0)	0	2 (22.2)	0
Insomnia	3 (27.3)	0	1 (9.1)	0	2 (16.7)	0	2 (18.2)	0	1 (16.7)	0	1 (11.1)	0
Rash	3 (27.3)	0	0	0	0	0	1 (9.1)	0	0	0	0	0
Nausea	2 (18.2)	0	5 (45.5)	0	3 (25.0)	0	2 (18.2)	0	1 (16.7)	1 (16.7)	0	0
Pyrexia	2 (18.2)	0	4 (36.4)	0	3 (25.0)	0	1 (9.1)	0	2 (33.3)	0	2 (22.2)	0
Asthenia	2 (18.2)	0	3 (27.3)	2 (18.2)	0	0	0	0	0	0	0	0
Peripheral sensory neuropathy	2 (18.2)	0	1 (9.1)	0	2 (16.7)	0	0	0	1 (16.7)	0	1 (11.1)	1 (11.1)
Hypoalbuminemia	1 (9.1)	1 (9.1)	3 (27.3)	2 (18.2)	4 (33.3)	0	0	0	0	0	2 (22.2)	1 (11.1)
Cardiac failure[b]	1 (9.1)	0	3 (27.3)	2 (18.2)	2 (16.7)	2 (16.7)	1 (9.1)	1 (9.1)	0	0	0	0
Hypokalemia	1 (9.1)	1 (9.1)	2 (18.2)	1 (9.1)	1 (8.3)	0	2 (18.2)	1 (9.1)	2 (33.3)	1 (16.7)	1 (11.1)	1 (11.1)
Hypercholesterolemia	1 (9.1)	0	1 (9.1)	0	3 (25.0)	1 (8.3)	1 (9.1)	1 (9.1)	1 (16.7)	0	2 (22.2)	1 (11.1)
Increased blood alkaline phosphatase	1 (9.1)	1 (9.1)	1 (9.1)	0	2 (16.7)	0	1 (9.1)	0	0	0	3 (33.3)	0
Hyponatremia	1 (9.1)	1 (9.1)	0	0	1 (8.3)	0	0	0	1 (16.7)	0	1 (11.1)	1 (11.1)
Decreased appetite	0	0	4 (36.4)	0	4 (33.3)	0	1 (9.1)	0	1 (16.7)	0	2 (22.2)	0

Hypertriglyceridemia	0	0	1 (9.1)	0	3 (25.0)	0	1 (9.1)	0	0	0	2 (22.2)	1 (11.1)
Increased blood creatinine	0	0	1 (9.1)	0	1 (8.3)	0	1 (9.1)	1 (9.1)	1 (16.7)	0	3 (33.3)	0
Hyperglycemia	0	0	1 (9.1)	0	1 (8.3)	0	1 (9.1)	0	1 (16.7)	1 (16.7)	1 (11.1)	0
Syncope	0	0	1 (9.1)	1 (9.1)	1 (8.3)	1 (8.3)	2 (18.2)	2 (18.2)	0	0	0	0
Acute kidney injury	0	0	1 (9.1)	0	0	0	0	0	0	0	1 (11.1)	1 (11.1)
Nephrotic syndrome	0	0	0	0	0	0	0	0	1 (16.7)	1 (16.7)	0	0
Acute respiratory distress syndrome	0	0	0	0	0	0	0	0	0	0	1 (11.1)	1 (11.1)

TEAE, treatment-emergent adverse event; D-VCd, daratumumab subcutaneous plus bortezomib/cyclophosphamide/dexamethasone; VCd, bortezomib/cyclophosphamide/dexamethasone.

[a]The safety population included patients who received ≥1 administration of study treatment.

[b]Includes overall and congestive cardiac failure.

Supplementary References

1. Palladini G, Sachchithanantham S, Milani P, Gillmore J, Foli A, Lachmann H, Basset M, Hawkins P, Merlini G, Wechalekar AD (2015) A European collaborative study of cyclophosphamide, bortezomib, and dexamethasone in upfront treatment of systemic AL amyloidosis. Blood 126:612–615. https://doi.org/10.1182/blood-2015-01-620302
2. Sidana S, Dispenzieri A, Murray DL, Go RS, Buadi FK, Lacy MQ, Gonsalves WI, Dingli D, Warsame R, Kourelis T, Muchtar E, Hayman SR, Kapoor P, Kyle RA, Leung N, Rajkumar SV, Gertz MA, Kumar SK (2020) Revisiting complete response in light chain amyloidosis. Leukemia 34:1472–1475. https://doi.org/10.1038/s41375-019-0664-9
3. Muchtar E, Dispenzieri A, Leung N, Lacy MQ, Buadi FK, Dingli D, Hayman SR, Kapoor P, Hwa YL, Fonder A, Hobbs M, Gonsalves W, Kourelis TV, Warsame R, Russell SJ, Lust JA, Lin Y, Go RS, Zeldenrust SR, Kyle RA, Rajkumar SV, Kumar SK, Gertz MA (2019) Optimizing deep response assessment for AL amyloidosis using involved free light chain level at end of therapy: failure of the serum free light chain ratio. Leukemia 33:527–531. https://doi.org/10.1038/s41375-018-0258-y
4. Manwani R, Cohen O, Sharpley F, Mahmood S, Sachchithanantham S, Foard D, Lachmann HJ, Quarta C, Fontana M, Gillmore JD, Whelan C, Hawkins PN, Wechalekar AD (2019) A prospective observational study of 915 patients with systemic AL amyloidosis treated with upfront bortezomib. Blood 134:2271–2280. https://doi.org/10.1182/blood.2019000834
5. Kastritis E, Palladini G, Minnema MC, Wechalekar AD, Jaccard A, Lee HC, Sanchorawala V, Gibbs S, Mollee P, Venner CP, Lu J, Schönland S, Gatt ME, Suzuki K, Kim K, Cibeira MT, Beksac M, Libby E, Valent J, Hungria V, Wong SW, Rosenzweig M, Bumma N, Huart A, Dimopoulos MA, Bhutani D, Waxman AJ, Goodman SA, Zonder JA, Lam S, Song K, Hansen T, Manier S, Roeloffzen W, Jamroziak K, Kwok F, Shimazaki C, Kim JS, Crusoe E, Ahmadi T, Tran NP, Qin X, Vasey SY, Tromp B, Schecter JM, Weiss BM, Zhuang SH, Vermeulen J, Merlini G, Comenzo RL (2021) Daratumumab-based treatment for immunoglobulin light-chain amyloidosis. N Engl J Med 385:46–58. https://doi.org/10.1056/NEJMoa2028631
6. Palladini G, Dispenzieri A, Gertz MA, Kumar S, Wechalekar A, Hawkins PN, Schonland S, Hegenbart U, Comenzo R, Kastritis E, Dimopoulos MA, Jaccard A, Klersy C, Merlini G (2012) New criteria for response to treatment in immunoglobulin light chain amyloidosis based on free light chain measurement and cardiac biomarkers: impact on survival outcomes. J Clin Oncol 30:4541–4549. doi:10.1200/JCO.2011.37.7614
7. Palladini G, Hegenbart U, Milani P, Kimmich C, Foli A, Ho AD, Vidus Rosin M, Albertini R, Moratti R, Merlini G, Schonland S (2014) A staging system for renal outcome and early markers of renal response to chemotherapy in AL amyloidosis. Blood 124:2325–2332. doi:10.1182/blood-2014-04-570010
